# Simple Use of the Suppository Type Povidone-Iodine Can Prevent Infectious Complications in Transrectal Ultrasound-Guided Prostate Biopsy

**DOI:** 10.1155/2009/750598

**Published:** 2009-04-23

**Authors:** Dong Soo Park, Jong Jin Oh, Jin Ha Lee, Woong Ki Jang, Young Kwon Hong, Sung Keun Hong

**Affiliations:** ^1^Department of Urology, College of Medicine, Pochon CHA University, Sungnam 463-712, South Korea; ^2^Department of Clinical Pathology, College of Medicine, Pochon CHA University, Sungnam 463-712, South Korea

## Abstract

*Purpose*. To determine the effect of simple use of suppository povidone-iodine on infectious complications after transrectal ultrasonography-guided biopsy of the prostate. *Methods*. All 481 patients are included and received antibiotic prophylaxis. Among them, 360 patients received povidone-iodine suppository (Gynobetadine; 200 mg) immediately prior to biopsy and 121 patients did not. Infectious complications were classified. To evaluate bactericidal effects, we counted bacterial colonies in the rectum, harvested from a rectal swab before insertion of the suppository and after biopsy. Aliquots of the suspended bacterial strains were added to Mueller-Hinton agar medium for incubation. Colony counts were determined. *Results*. Infectious complications developed in 1 case (0.3%) in the rectal preparation group (Group 1) and in 8 cases (6.6%) in the nonrectal preparation group (Group 2). One in Group 1 had a fever without sepsis. Two patients had sepsis and six had fever without sepsis in Group 2. Rectal preparation was a statistically significant risk factor influencing the development of infectious complications. In vitro experiments, the mean number of colony-forming units decreased 99.9% after the rectal povidone-iodine preparation. *Conclusions*. All through the biopsy, povidone-iodine melted into the rectum and decreased the bacterial colony count. Simple use of povidone-iodine suppository before prostate biopsy minimizes the risk of infectious complications.

## 1. Introduction

Since the introduction of PSA as a marker in the diagnosis of prostate cancer, the rate of biopsies taken from the prostate has increased dramatically. Although prostate biopsies are generally considered a safe procedure, complications secondary to biopsy are some of the most common adverse events encountered in practice. Usually minor and self-limiting, they are occasionally serious and even fatal. A major problem of the transrectal approach is the risk of infection, leading to febrile reactions, epididymitis, prostatitis, sepsis, and even death. Currently, many urologists use prophylactic antibiotic therapy to minimize the infectious complications after biopsy, but these therapies do not completely eliminate the incidence of infection [1, 2]. For this reason, a rectal preparation has been used as a cleansing technique before biopsy to reduce infectious complications [1–3]. However, the effect of a rectal preparation applied before biopsy has not yet been validated. We therefore determined the effect of povidone-iodine suppository (Gynobetadine) prophylaxis on infectious complications after transrectal needle biopsy of the prostate.

## 2. Material and Methods

This study was performed on 481 patients in our hospital from January 2004 to December 2007 who had indications for a prostate biopsy, including an elevated PSA, hypoechoic lesions on ultrasonography, or abnormal digital rectal findings.

All patients received antibiotic prophylaxis consisting of a single intravenous injection of a 3rd generation cephalosporin just before biopsy and oral administration of cefixime (100 mg twice daily) at morning of day of biopsy and then continued for 5 days thereafter. Between April 2005 and December 2007, 360 patients (group 1) received a povidone-iodine suppository (Gynobetadine; 200 mg) just before biopsy, and between January 2004 and March 2005 121 patients (group 2) did not receive Gynobetadine. All patients were provided with informed consent before the biopsy. Transrectal ultrasound (TRUS-) guided prostate biopsies were performed without anesthesia in an outpatient setting by experienced urology doctors. 12 cores were obtained in the same way between two groups. An Aloka Prosound SSO-4000 ultrasonography unit and a transrectal 7.5 MHz biplane probe were used during the procedure. Biopsies were taken with an 18G ASAP automatic biopsy needle (SACN Biopsy Needle). At the one week followup visit, any complications after the biopsy were evaluated and recorded.

Infectious complications were classified as sepsis, fever (temperature higher than 38°C) without sepsis, and other clinical infections. Sepsis was defined as two or more of the following conditions as a result of infection: body temperature >38°C or <36°C, heart rate >90 beats per minute, respiration rate >20 breaths per minute or arterial partial pressure of carbon dioxide <32 mmHg, and white blood cell count >12 000 cells/cm^3^ or <4000 cells/cm^3^, or >10% immature (band) forms [4]. Noninfectious complications were evaluated and classified as hematuria, rectal bleeding, and perineal pain.

To evaluate the bactericidal effects of Gynobetadine, we counted bacterial colonies in the rectum that had been harvested from rectal swab before the insertion of Gynobetadine and after biopsy. Colony counts were determined by agar dilution. Bacterial strains of the rectal swab were suspended and diluted in sterile saline, and aliquots of this suspension were added to Mueller-Hinton agar medium. After overnight incubation, the colony counts were determined.

## 3. Results

The mean ages of groups 1 and 2 patients were 64.6 years (range, 29–88 years) and 65.7 years (range, 31–96 years), respectively. The PSA levels in groups 1 and 2 were 6.45 ng/mL and 7.34 ng/mL, , respectively. The patient demographic data and complications are shown in Table 1. There was no statistically significant difference between groups 1 and 2 with respect to demographic data.

**Figure 1:**
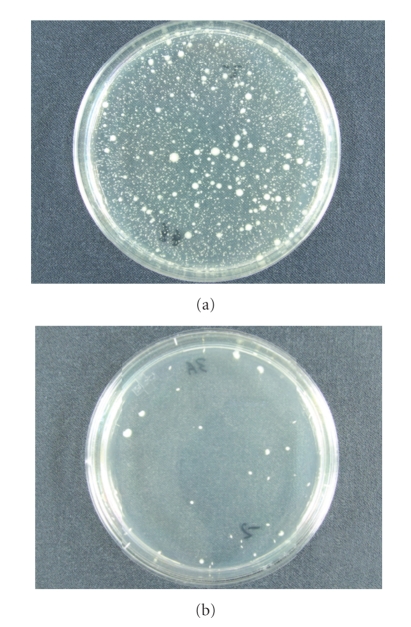
In in vitro experiments (a) bacterial colony plate in an agar dilution before insertion of povidone-iodine. (b) Bacterial colony plate in an agar dilution after biopsy; the mean number of colony-forming units decreased 99.9% after rectal preparation.

Infectious complications developed in 8 cases (6.6%) in the nonrectal preparation group (group 2) and in 1 case (0.3%) in the rectal preparation group (group 1). Among eight infectious complications in the nonrectal preparation group, two patients had sepsis and six patients had fever without sepsis. Among the eight patients, four patients developed an acute bacterial prostatitis and one patient developed an acute epididymitis. One patient from group I complained of a febrile sensation one day after biopsy, which resolved spontaneously. A single rectal preparation using Gynobetadine significantly lowered the risk of development of infectious complications (*P* = .001). In in vitro experiments, the mean number of colony-forming units decreased 99.9% after rectal preparation (Table 2). We found that the bacterial colony count before rectal preparation ranged from 7.0 × 10^3^ to 7.0 × 10^5^, but decreased to <3.0 × 10^1^ after biopsy. Figure 1 shows the bactericidal effects of povidone-iodine, and it is clear that the bacterial count was significantly reduced after the insertion of Gynobetadine. The Gynobetadine preparation statistically decreased the colony count (*P* = .002).

Noninfectious complications (classified as hematuria, rectal bleeding, and perineal pain) developed in 22 cases (18.2%) in the nonrectal preparation group and in 29 cases (8.1%) in the rectal preparation group. The noninfectious complications were self-limiting and did not require intervention or transfusion. No patient developed urinary retention after the procedure. There was no statistically significant difference in terms of noninfectious complications.

## 4. Discussion

Transrectal prostate biopsy with ultrasonography is the gold standard in the diagnosis of prostate cancer. Most men undergoing prostate biopsy are otherwise healthy. The procedure, however, is not harmless. Transient side events, such as local pain, hematuria, hemospermia, dysuria, and rectal bleeding have been reported in a large number of patients [5, 6]. Bacteriuria occurs in 20–53% and bacteremia in as many as 73% of patients following a biopsy of the prostate [7, 8], and rectal bleeding has been reported in 75% of such patients [2–4]. Fever associated with genitourinary symptoms is described in 3–10% and septicemia in <5% of patients following a biopsy of the prostate [7–9]. Antimicrobial agents lower the incidence of postbiopsy infectious complications [1, 9, 10]. Most urologists agree in the necessity of antibiotic prophylaxis, but the timing, duration of the regimen, and the route of administration remain controversial [11]. In most studies, an antibiotic regimen of at least 3 days has been used [12].

Despite the use of prophylactic antibiotics, the development of infectious complications after biopsy remains possible, although its incidence is low [1, 13, 14]. For this reason, a cleansing rectal enema has been used by 81% of urologists as a means to reduce infectious complications [15]. Most investigators believe that rectal preparation reduces the rate of bacteremia [16, 17]. In a prospective randomized study, Lindert et al. [8] proposed that bacteremia might be minimized by a prebiopsy enema. In their study, bacteremia following prostate biopsy occurred in 4% (1 of 25) of patients who had prebiopsy enemas compared with 28% (7 of 25) of those who did not. This study therefore provided a theoretical basis for using a prebiopsy rectal preparation for the prevention of infectious complications.

Povidone-iodine also may be used to prepare the rectum for transrectal biopsy of the prostate intraoperatively. The use of povidone-iodine is well known in clinical medical practice. Its use in dermatology and surgery as a disinfectant is ubiquitous and is based on the fact that povidone-iodine has broad-spectrum antimicrobial activity [18], including viruses [19], fungi [20], and parasites, as well as anti-inflammatory activity [21]. Povidone-iodine is an antiseptic solution extensively used in different concentrations.

Brown et al. [16] reported that a povidone-iodine enema provided a safe and effective means for preventing bacteremia and bacteriuria and Melekos [17] showed that parenteral piperacillin in combination with a povidone-iodine enema significantly reduces the incidence of infectious complications. Khan et al. [22] reported that intraoperative preparation of the rectum with a povidone-iodine-saturated gauze during transrectal biopsy of prostate decreased the incidence of bacteriuria and bacteremia. Akay et al. [23] reported that lavage with Betadine prior to transrectal prostate biopsy is adequate in the prevention of infectious complications.

Huang et al. [24] reported that a phosphate-based enema combined with povidone-iodine is effective in reducing postprostate biopsy infectious complications (9.23 versus 0%).

A povidone-iodine suppository (Gynobetadine; 200 mg) is more effective than a povidone-iodine enema, because the contact surface between the rectal mucosa of the prostate area is more constant during the biopsy procedure than with the enema. With the enema technique, the solution of povidone-iodine is retained in the dependent portion of the rectum and may not effectively bathe the rectal mucosa of the prostatic area. A povidone-iodine suppository has better patient acceptance than an enema.

There have been some reports that have shown the effects of povidone-iodine in the treatment of infectious complications [22]. However, there are no studies that have verified the bactericidal effects of povidone-iodine in in vitro experiments. For this reason, we counted bacterial colonies in the rectum that had been harvested from rectal swabs before the insertion of povidone-iodine and after biopsy to evaluate the bactericidal effects of povidone-iodine. We found that the bacterial colony counts before the rectal preparation were decreased dramatically after biopsy. These local antiseptic precautions seem worthwhile. With the large reduction in microorganisms, contamination is likely to be considerably reduced.

Periprostatic local anesthesia for transrectal ultrasound-guided prostate biopsy is known to reduce pain but increases the incidence of bacteriuria and bacteremia [25]. Using our technique with povidone-iodine, local anesthesia for prostate biopsy is effective and safe for those in which it is necessary.

According to the data obtained in the present study, it is apparent that a Gynobetadine rectal preparation is reliable in preventing infection. Other anti-infectious enemas increase patient cost and discomfort [26]. The cost of Gynobetadine is very low (approximately US $0.50), and it is comfortable to patients by reason of quick and easy delivery. And it is better tolerated to the patients. After our prospective randomized trial with iodine suppository considered, we conclude that the povidone-iodine suppository is important to protect against infectious complications in patients undergoing biopsy of the prostate.

## 5. Conclusions

Simple rectal preparation using a povidone-iodine suppository was a statistically significant risk factor for infectious complications after prostate biopsy in our study.

Thus, we recommend a povidone-iodine suppository (200 mg) rectal preparation before prostate biopsy because of its effectiveness in controlling rectal bacterial colony-forming units and species, its very low cost, and its availability.

## Figures and Tables

**Table 1:** Demographic data of patients.

	Group 1	Group 2
Number of patients	360	121
Mean age (years)	64.6 (29–88)	65.7 (31–96)
Average PSA (ng/mL)	6.45	7.34
Prostate cancer diagnosis	104 (29%)	30 (25%)
Infective complications*	1	8
* *Sepsis	0	2
* *Fever without sepsis	1	6
Noninfective complications^†^	29	22
* *Hematuria	17	13
* *Rectal bleeding	5	4
* *Perineal pain	7	5

*P = .001;  ^†^ *P* = .391.

**Table 2:** Colony counts before insertion of povidone-iodine and after biopsy.

Plate no.	Before	After
1	1.19 × 10^5^	5.0 × 10^2^
2	1.93 × 10^4^	< 3.0 × 10^1^
3	1.09 × 10^4^	< 3.0 × 10^1^
4	7.0 × 10^3^	3.6 × 10^1^
5	7.0 × 10^5^	< 3.0 × 10^1^
6	7.4 × 10^4^	4.0 × 10^2^

*P = .002.
